# Structure Design and Working Characteristics Analysis of Direct-Drive Giant Magnetostrictive Injector

**DOI:** 10.3390/mi13101721

**Published:** 2022-10-12

**Authors:** Zhaoqi Zhou, Zhongbo He, Guangming Xue, Jingtao Zhou, Ce Rong, Guoping Liu

**Affiliations:** 1 Department of Vehicle and Electrical Engineering, Army Engineering University of PLA, Shijiazhuang 050001, China; 2 Mechanical Engineering and Automation College, Fuzhou University, Fuzhou 350116, China

**Keywords:** direct-drive injector, giant magnetostrictive material, giant magnetostrictive injector, numerical model, experiment verification, simulation analysis, operating characteristic

## Abstract

At present, the research of electronically controlled injectors is mostly limited to the non-direct drive structure. Although the research on the direct drive structure is involved, it mostly stays in the conceptual machine or simulation stage. In this paper, based on the direct-drive structure, the giant magnetostrictive material is used as the energy conversion material, the prototype of the direct-drive giant magnetostrictive fuel injector is designed and manufactured, and the experimental test system and AMESim simulation model are built. By means of experiment and simulation, the injection characteristics of Giant magnetostrictive injector (GMI) are tested. It is found that the minimum single injection quantity of GMI is 5.9 mm^3^ under the condition of 30 MPa rail pressure, which shows high injection accuracy. The experimental results are in good agreement with the simulation results under different driving pulse widths and voltages. When the driving pulse width is not less than 650 µs, the relative errors are all less than 5%, which verifies the effectiveness of the simulation model. The injection performance of GMI is analyzed. The results show that this injector has a stable injection performance, fast response speed (the shortest injection pulse width is about 200 µs), and the injection process can be completed five times in 5 ms.

## 1. Introduction

Diesel engines are widely used in industry, agriculture, transportation and national defense construction because of their high thermal efficiency and good adaptability [1]. However, since the 1970s, due to the exhaustion of fossil energy and the aggravation of environmental pollution, countries have successively issued very strict emission regulations. Aiming at the problem of vehicle exhaust emission, China has successively issued the fifth and sixth standards of vehicle emission according to its national conditions. As of 1 July 2021, there are more than 11 million heavy-duty diesel vehicles in China, accounting for only 4.4% of the total number of vehicles in China, but their emissions of nitrogen oxides and particulate matter, respectively, amount to 85% and 65% of the total vehicle emissions. Compared with the national standard five, the requirements of the national standard six for heavy diesel vehicles are further tightened, and the limits of nitrogen oxides and particulate matter are reduced by 77% and 67%, respectively [2]. The development of the diesel engine is facing new and huge challenges. How to further improve the fuel efficiency of the diesel engine and develop a “green diesel engine” that meets the requirements of high power, low fuel consumption, and low emission has become a key research focus of experts and scholars in the industry.

**Figure 1:**
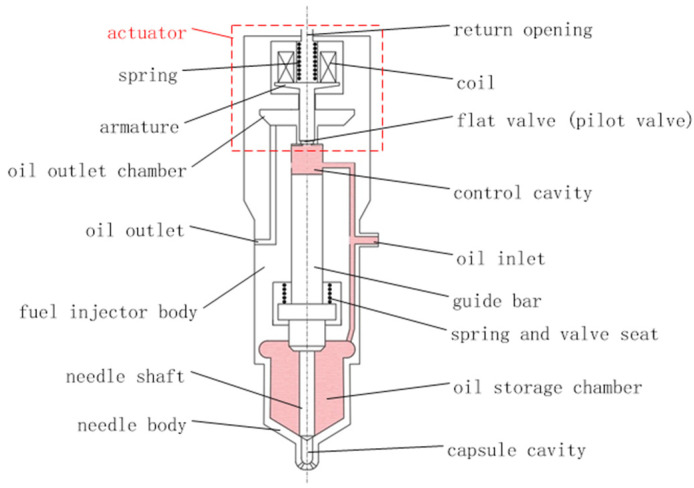
Schematic diagram of injector structure.

The fuel system is considered as the heart of diesel engine, and the fuel injector, as the terminal of the fuel system, is the core component of the whole fuel system, which determines the in-cylinder fuel injection process, and its fuel injection performance has a profound impact on the combustion process of diesel engine. According to the movement and hydraulic characteristics of each component, the electronically controlled injector can be divided into two parts: the actuator and the chamber-pipe-valve, as shown in Figure 1 [3].

**Figure 2:**
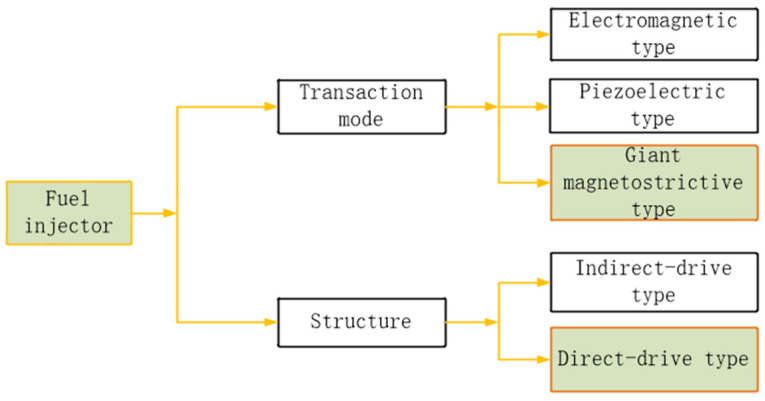
Classification of injectors.

According to the different transducer materials used in the actuator, the injectors can be divided into electromagnetic, piezoelectric, and giant magnetostrictive materials (GMM) [4,5] injectors, and according to the different driving modes of needle valves, the injectors can be divided into non-direct-drive and direct-drive injectors, as shown in Figure 2.

Among them, the giant magnetostrictive actuator (GMA) [6,7], developed by using GMM as a transducing material, shows excellent driving characteristics and shows great application prospects in the field of fuel injection.

Compared with piezoelectric materials and traditional magnetostrictive materials, giant magnetostrictive materials have excellent characteristics, such as large magnetostrictive strain, high magneto-mechanical conversion efficiency, high energy density, etc., and show remarkable application advantages in the field of actuators. The performance indexes of the three materials are shown in the Table 1 [8]:

Many scholars have developed various types of GMA based on the excellent characteristics of GMM and applied this GMA to the field of fuel injection, which greatly improved the fuel injection performance of fuel injectors.

Hirohisa Tanaka [9,10] of Yokohama National University designed an injector by superimposing the output displacements of two GMM rods. When the input voltage is 24 V and the common rail pressure is 160 MPa, the output displacement of GMA can reach 50 µm, and the injection rate is 30 mm^3^/ms, which shows a good linear relationship with the input current. However, the displacement superposition device is complex in structure and large in volume. In order to magnetize GMM rods to the maximum extent, the designed excitation coil turns more, which further increases the energy loss of the device and reduces the working stability of GMM rods.

Xue Guangming [11] of the Army University of Engineering designed a zero-offset GMA and applied it to the fuel injector. Driven by 24 V voltage, GMA output displacement is about 36 µm; under the starting voltage of 80 V, the shortest injection pulse width of giant magnetostrictive injector GMI [12] is less than 0.2 ms, the maximum injection rate can reach 75 mm^3^/ms, and the minimum single injection quantity is about 4.5 mm^3^, which can realize the high-precision control of injection quantity as well as large-flow injection.

Bin Xu [13] of the Anhui University of Science and Technology, based on GMM’s magnetostriction effect, designed a direct-drive fuel injector conceptual machine with the help of flexible hinge amplification mechanism. The simulation results show that when the common rail oil pressure is 140 MPa, the needle valve lift of the injector can reach 0.4 mm, and the nozzle flow rate is about 7 mg/ms, showing superior injection characteristics.

In addition, the giant magnetostrictive fuel injector has been actively explored in the literature [14,15,16,17,18,19,20] and very fruitful results have been achieved.

GMM is used as the driving material of the fuel injector, which greatly improves the fuel injection performance of the fuel injector. However, the related research mostly stays in the non-direct drive structure, and its inherent problems, such as long hydraulic circuit and lagging response, are hard to overcome. However, there is little research on direct-drive injectors, and most scholars only design and simulate the conceptual engine, but do not manufacture the prototype, and there is no corresponding experimental verification.

With the progress of manufacturing technology and the continuous enrichment of fuel injector structure design theory, the development of a direct-drive fuel injector has become a reality. In this paper, the direct-drive GMI is designed independently, and related research work is carried out. Firstly, based on the driving demand of fuel injector and the output characteristics of GMA, the structure design of GMI is completed, and the prototype is made. Secondly, combined with the GMI structure principle and working process, the GMI overall model, which is suitable for AMESim simulation platform, is established and the experimental test system is established. The correctness of the numerical model is completed through the experimental test. Finally, the working characteristics of GMI are analyzed by AMESim simulation software, including the injection accuracy, response characteristics, and injection rate shape of GMI.

## 2. Structure Design and Theoretical Model

### 2.1. GMI Structure Design

**Figure 3:**
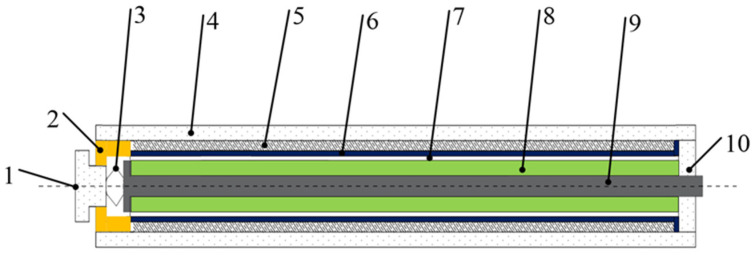
GMA structure diagram. (1) Tightening bolt; (2) Upper end cover; (3) Disc spring; (4) Shell; (5) Excitation coil; (6) Coil skeleton; (7) Air gap; (8) GMM cylinder; (9) “T” plunger; (10) Lower end cover.

In the direct-drive injector, the needle valve is directly controlled by the actuator. To ensure the working stability of the injector, its working mode should be powered off and normally closed. Therefore, when the actuator is energized, it should output upward displacement to open the needle valve. Some scholars apply strong bias magnetic field to cause GMM to reach the maximum elongation, and when energized, the coil generates reverse magnetic field, which shortens GMM. The design scheme can meet the working requirements of fuel injectors to some extent, but this structural design scheme increases the volume of GMA, and it is difficult to accurately grasp the critical point of the maximum displacement output when the reverse magnetic field is applied, and it is easy to cause the abnormal fluctuation of GMA output displacement near the critical point. A reasonable scheme is to design GMA by matching T-shaped plungers with the GMM cylinder, as shown in Figure 3 [21].

**Figure 4:**
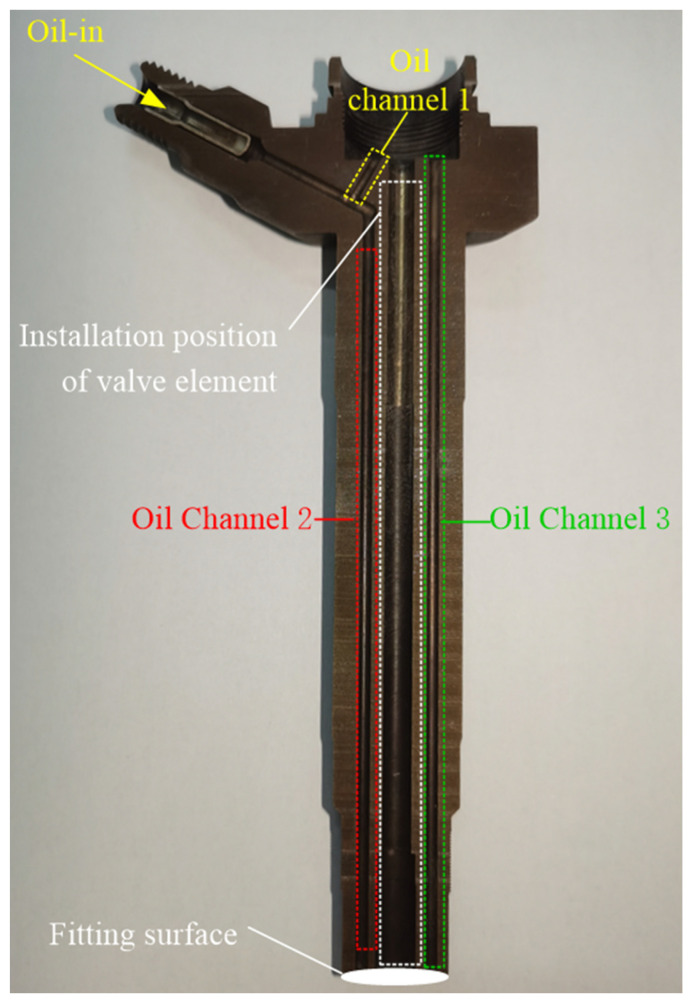
Partial sectional view of electromagnetic injector.

The pipeline–oil chamber–valve part involves complicated mechanical and hydraulic characteristics. On the one hand, this part is combined with GMA part to realize the transmission of power from actuator output rod to injector needle valve. On the one hand, it communicates with the high-pressure common rail fuel and directly acts on the conical surface of the needle valve, which puts forward higher requirements for its tightness and rigidity. Considering the factors such as processing cost and development cycle, this paper utilized the existing mature products on the market, reasonably modifying them to meet the working requirements of direct-drive injectors. The local structure of an electric electromagnetic injector is shown in Figure 4. After the high-pressure fuel enters the injector through the oil inlet, part of the fuel flows into the control chamber through the oil channel 1, and part of the fuel flows to the nozzle through the oil channel 2. The fuel injector body and the nozzle form the mating surface shown in Figure 4. When the fuel flows to the mating surface through the oil channel 2, part of the fuel leaks into the oil channel 3 at the mating surface, and most of the fuel flows into the nozzle.

**Figure 5:**
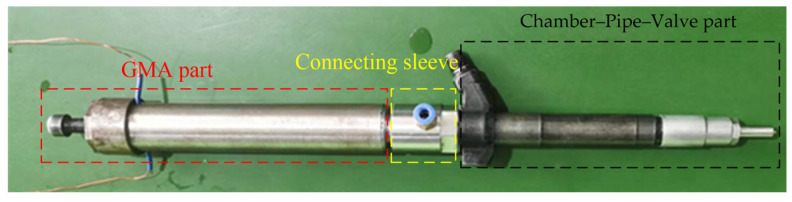
GMI prototype.

A direct-drive GMI has been manufactured independently, and relevant research has been carried out. The GMI prototype is shown in Figure 5, and its working principle is as follows: when the power is not turned on, the GMA output rod, the needle valve rod and the needle valve are in close contact due to the pre-tightening force, and the needle valve is kept closed at this time. After power-on, GMA outputs displacement, the GMI needle valve synchronously lifts under the action of oil, and the injector starts to inject oil. After the power is cut off, the GMA output rod pushes the needle valve to overcome the oil pressure to close the nozzle under the pre-tightening force.

### 2.2. GMA Model

The research of GMA is an interdisciplinary subject in the field of mechatronics, which makes the GMA model complicated and computationally intensive. Considering that the GMA model is combined with the cavity–pipeline–valve part model, the GMA model should not be too complicated. Therefore, according to the working sequence of GMA, the GMA weak coupling model is here established with voltage as input and GMA magnetostrictive force as output, and each link is connected by a single physical field [18].

The relationship between current I and voltage U in the coil can be obtained by equating the coil to a series circuit of resistor *R_L_* and unimpeded *L* inductor.

(1) $$R_{L}I+L\dot{I}=U$$

The magnetic field strength *H* on GMM cylinder can be easily obtained by using magnetic circuit model.

(2) $$H=C\frac{NI}{l}$$

In this formula, *C* is the proportional coefficient of reluctance, *N* is the number of coil turns, and *l* is the length of GMM barrel.

The magnetic flux *φ* in the magnetic circuit can be expressed as

(3) $$\varphi =\frac{NI}{r}$$

where *r* is the total reluctance of the magnetic circuit.

(4) φ=BS

where *S* is the cross-sectional area of GMM cylinder.

In magnetic media, magnetic induction *B*, magnetic field *H*, and magnetization *M* have the following relationship:

(5) $$B={µ}_{0}\left(H+M\right)$$

where ${µ}_{0}$ is permeability of vacuum.

From the quadratic domain rotation model, the magnetostrictive strain *λ* of GMM cylinder can be obtained as follows:

(6) $$\lambda =\frac{3\lambda_{S}}{2M_{S}^{2}}M^{2}$$

where *λ_S_* is the saturated magnetostriction strain and MS is the saturated magnetization.

The magnetostrictive force *F* of GMM cylinder can be expressed as:

(7) F=λES

where *E* is the elastic modulus of GMM.

### 2.3. Model of Chamber–Pipe–Valve

The internal hydraulic system of the injector plays an important role in the injection characteristics of the injector, profoundly affecting the action process of the needle valve and the flow of fuel. The hydraulic system model mainly includes the cavity model, pipeline model, valve element model, leakage module model, etc. [22,23].

#### 2.3.1. Cavity Model

The volume of the cavity has an important influence on the fuel injection characteristics but has nothing to do with its specific structure size. Therefore, in the electronically controlled injector, the oil storage chamber, pressure chamber, and so on can be simplified as a centralized chamber. For the concentration chamber, the relationship between fuel flow rate and fuel flow rate can be expressed as:

(8) $$\frac{dp}{dt}=\frac{G\left(p\right)\sum_{i}q_{i}\left(p_{i}\right)}{\sum_{i}v_{i}+v_{0}}$$

where *G* is the bulk elastic modulus of fuel; *p* is the outlet pressure in the chamber; *p_i_* is the outlet pressure in the chamber; *q_i_* is the flow rate of each inlet in the chamber; *v_i_* is the irregular volume in the cavity, which can be ignored when the value is small; and *v*_0_ is the fixed dead volume of the concentration chamber.

#### 2.3.2. Pipeline Model

The common rail pipe connected with the oil inlet of the injector and the oil channel inside the direct-drive injector designed in this paper belongs to the pipeline structure. Ignoring the pressure fluctuation and fuel temperature change, the equations of mass conservation and the momentum conservation of the pipeline can be obtained.

(9) $$\frac{\partial \rho }{\partial t}+\frac{\partial \rho u}{\partial x}=0$$

(10) $$\frac{\partial \rho u}{\partial x}+\frac{\partial \rho u^{2}+p}{\partial x}+h_{f}=0$$

where *ρ* is the fuel density; *u* is the flow velocity; *p* is the pressure; and *h_f_* is a friction-related term.

#### 2.3.3. Valve Element Model

In the fuel injector, the control plunger and needle valve are kept under pressure in the working process. On the other hand, the output end of the “T” plunger in GMA is always in contact with the control plunger. Therefore, it is considered that the three keep synchronous motion.

(11) $$m_{1}\frac{d^{2}h}{dt^{2}}+c_{1}\frac{dh}{dt}=\sum F_{m}+\sum F_{h}+f$$

where *m*_1_ is the mass of the valve; *h* is valve displacement; *c*_1_ is the damping coefficient of valve movement; $\sum F_{m}$ is the mechanical resultant force of the valve; $\sum F_{h}$ is the resultant force of hydraulic pressure on the valve; and *f* is the magnetostriction force on the valve.

#### 2.3.4. Leak Module Model

When the injector is working at high pressure, there is oil leakage between the internal moving parts. The leakage flow rate *q_l_* can be expressed as:

(12) $$q_{l}=\left(p_{1}{}^{2}-p_{2}{}^{2}\right)\frac{\pi d_{p}r_{c}{}^{3}}{6\mu l}$$

where *p_1_* and *p_2_* are the oil pressure at both ends, respectively; *d_p_* is the diameter of the outer cylinder; *r_c_* is the radial clearance of the coupling parts; *μ* is the kinematic viscosity of oil; and *l* is the contact length between moving pairs.

#### 2.3.5. Throttle Model

Throttling action exists at the inlet, return and inlet of the fuel injector, which will have an important impact on the fuel injection characteristics of the fuel injector. The basic flow equation is:

(13) $$q_{o}=\{\begin{array}{c} C_{q}A\sqrt{\frac{2\left|\Delta p\right|}{\rho }}\sqrt{\frac{x_{gr}+1}{x_{gr}}}x\leq x_{gr}\\ C_{q}A\sqrt{\frac{2\left|\Delta p\right|}{\rho }}\sqrt{\frac{x+1}{x}},x>x_{gr} \end{array}$$

where *q_o_* is the output flow; *C_q_* is the flow coefficient; *A* is the effective flow cross-sectional area of the orifice; *ρ* is fuel density; *x_gr_* is the critical cavitation coefficient; and *x* is the cavitation coefficient, which is determined by the oil pressures *p*_1_ and *p*_2_ on both sides of the throttle.

(14) $$x=\{\begin{array}{c} \frac{p_{1}\;-\;p_{2}}{p_{2}\;+\;1.013\;\times \;10^{5}},\;p_{1}>p_{2}\\ \frac{p_{2}\;-\;p_{1}}{p_{1}\;+\;1.013\;\times \;10^{5}},\;p_{1}\leq p_{2} \end{array}\;$$

## 3. AMESim Simulation Model Building and Experimental Verification

In recent years, the vigorous development of simulation software has brought great convenience to the design of device structure and feasibility analysis and has gradually become a powerful research tool for experts and scholars. In this paper, AMESim simulation software is used to analyze the fuel injection performance of a direct-drive GMI.

### 3.1. Introduction to Software

AMESim (Advanced Modeling Environment for Performing Simulations of Engineering Systems) is an advanced modeling and simulation platform software for engineering systems [24]. Through simple component sub-models, complex models coupled with multiple physical fields can be conveniently and quickly built. It is widely used in the research and analysis of fuel injection, hydraulic systems, electromechanical systems, power transmission, and cooling systems and is an ideal choice for studying electronically controlled injectors. In addition, AMESim software has a unique way of defining magnetic materials, which can accurately describe the characteristics of GMM materials by defining related parameters, which provides strong support for the accurate simulation of GMI and effectively avoids the complexity of establishing and solving a GMM constitutive model.

### 3.2. Establishment of Direct-Drive GMI Simulation Model

**Figure 6:**
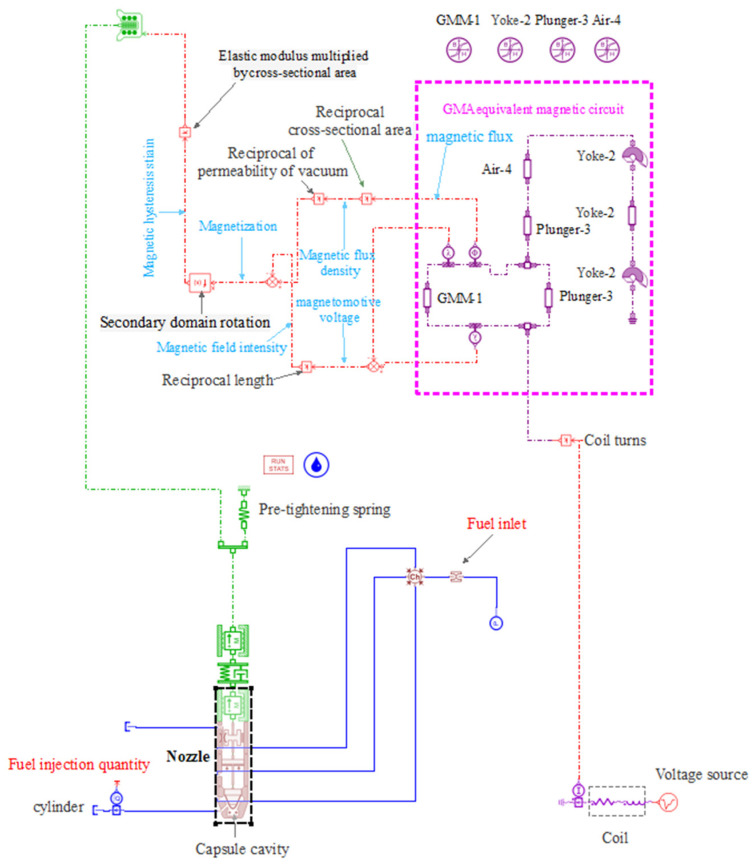
GMI simulation model building.

Based on GMI structure and GMI theoretical model, the AMESim simulation model as shown in Figure 6 is built.

The main elements involved in the simulation are shown in Table 2, and the main parameters of GMI are shown in Table 3.

### 3.3. Experimental Verification

**Figure 7:**
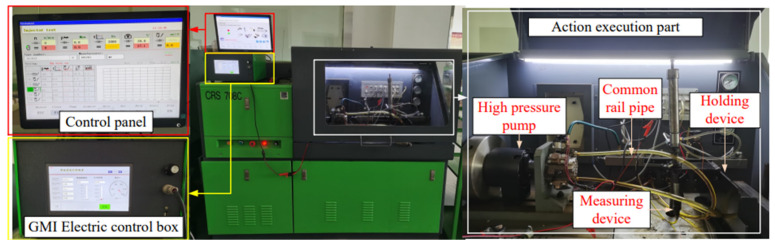
GMI experimental test system.

**Figure 8:**
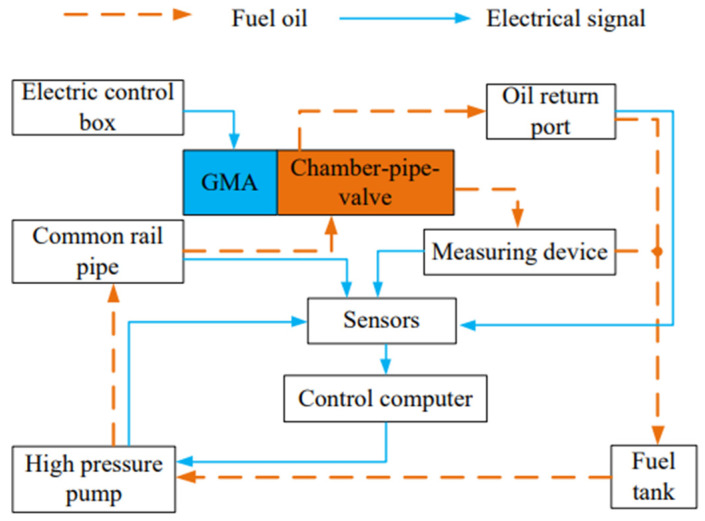
GMI experimental test principle.

A test system as shown in Figure 7 is set up. The system consists of a high-voltage common rail test platform, electronic control box, and GMI prototype. The model of a high-pressure common rail test platform is CRS-708C, which is composed of a main control computer, high-pressure oil pump, common rail pipe, fuel injection quantity test device, and other components and can provide common rail pressure of 0~200 MPa. The state parameters, such as oil pressure, temperature, high-pressure oil pump speed, etc., in the working process of fuel injector can be accurately detected and adjusted by computer. The electronic control box is GMI special power supply, and parameters, such as fuel injection pulse width and output voltage, can be set according to fuel injection requirements. The testing principle of GMI testing system is shown in Figure 8.

**Figure 9:**
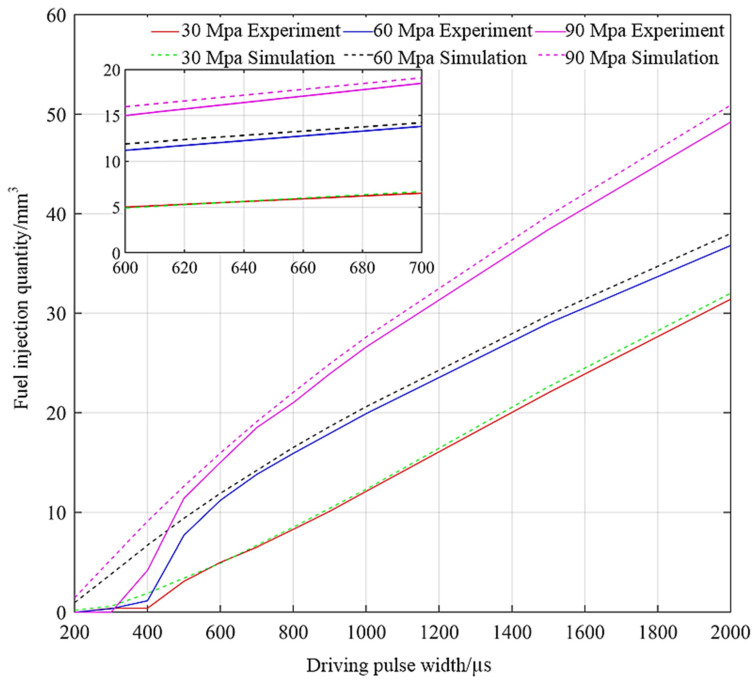
GMI single injection quantity under different driving pulse widths.

**Figure 10:**
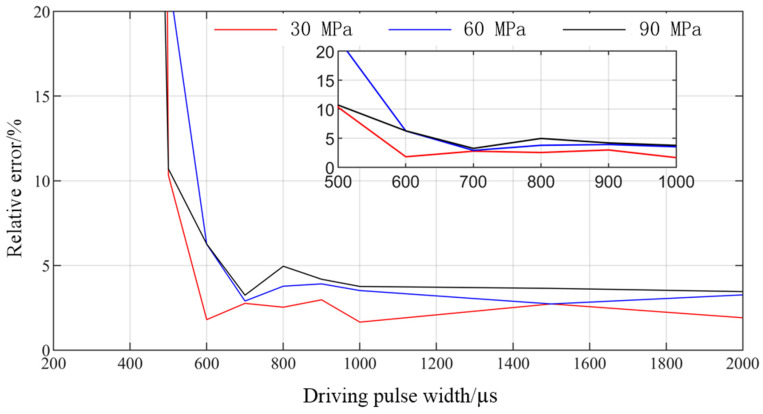
Relative error of DD-GMI single injection quantity under different driving pulse widths.

The variation trend of GMI with driving pulse width is obtained when the rail pressure is 30 MPa, 60 MPa, and 90 MPa, respectively. The experimental results, simulation results, and relative error distribution are shown in Figure 9 and Figure 10. It can be seen from Figure 9 that the simulation results are in good agreement with the experimental results. When the driving pulse width is larger than 600 µs, there is a good linear relationship between them and the driving pulse width, which verifies the correctness of the simulation model. The experimental results show that the minimum injection quantity of GMI is 5.9 mm^3^ under 24 V driving voltage, which shows good injection accuracy. The experimental fuel injection quantity is less than the simulated fuel injection quantity, which is caused by fuel leakage, fuel resistance, and other factors in the actual injection process. As can be seen from Figure 10, when the driving pulse width is 200 µs~650 µs, the relative error is large. This is because the inertia of the moving parts of the fuel injector is large and the driving voltage is small, so that the needle valve cannot rise quickly when the pulse width is small, resulting in small GMI injection quantity or even no injection. From 650 µs to 2000 µs, the relative errors are all less than 5%, which shows high calculation accuracy. With the increase of rail pressure, the injection quantity of direct-drive giant magnetostrictive injector (DD-GMI) increases, and at the same time, the relative error also increases. This is because the increase of rail pressure makes the fuel leakage of injector increase.

**Figure 11:**
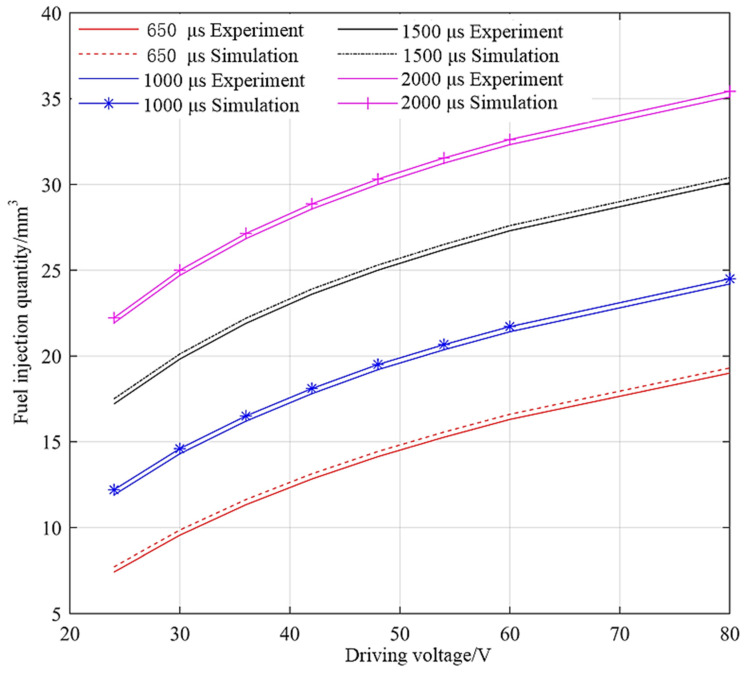
GMI single injection quantity under different driving voltages.

**Figure 12:**
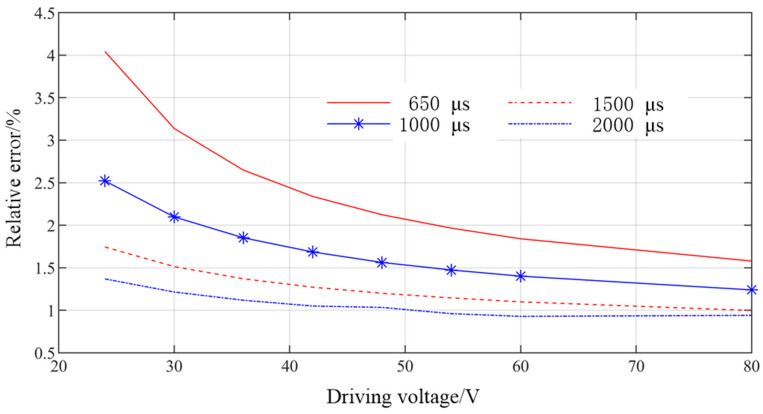
Relative error of DD-GMI single injection quantity under different driving voltages.

When the common rail pressure is 30 Mpa and the driving pulse width is larger than 650 µs, the single injection quantity of GMI under different driving voltages is measured. The experimental results, simulation results, and relative errors are shown in Figure 11 and Figure 12. As can be seen from Figure 11, the experimental results are in good agreement with the simulation results, and the experimental results are all smaller than the simulation results. With the increase of driving voltage, GMI injection quantity increases nonlinearly, which is mainly affected by the displacement of the needle valve. With the increase of driving pulse width, the injection quantity also increases. It can be seen from Figure 12 that when the pulse width is greater than 650 µs, the relative errors are all less than 5%. With the increase of driving voltage, the relative error decreases, because the larger needle valve displacement makes the leakage of fuel injector smaller under the same rail pressure. With the increase of driving pulse width, the relative error decreases. This is because for one injection process, the error is mainly introduced when the pulse width is short, while the influence of injection error on the relative error of the whole injection process is weakened when the pulse width is large.

## 4. GMI Fuel Injection Performance Analysis

In Section 3.3, the single injection quantity of GMI under different driving pulse widths and driving voltages was tested through experiments, and the correctness of the simulation model was verified. As the simulation analysis can more comprehensively analyze the fuel injection performance of the injector under the premise of low cost, this section provides an in-depth analysis of the fuel injection performance of DD-GMI with the help of the simulation model.

### 4.1. Large Pulse Width and Long Spray

**Figure 13:**
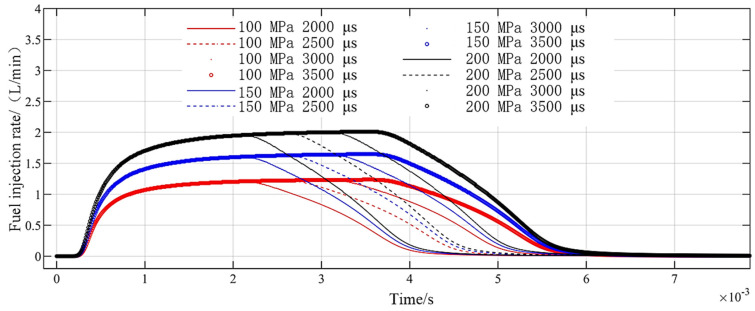
GMI injection rate under different big pulse widths.

The fuel injection rate is driven by square wave with amplitude of 24 V and pulse width of 2000 µs, 2500 µs, 3000 µs, and 3500 µs, and the rail voltage is set to 100 MPa, 150 MPa, and 200 MPa, respectively. The fuel injection rate of DD-GMI is shown in Figure 13. When the driving pulse width increases from 2000 µs to 3500 µs, the fuel injection duration of DD-GMI is prolonged, and at the same rail pressure, the fuel injection rate increases and decreases at the same speed, which indicates that DD-GMI has good working stability. Common rail pressure increased from 100 MPa to 200 MPa, and the maximum fuel injection rate increased from 1.2 L/min to 2.02 L/min.

### 4.2. Small Pulse Width and Short Jet

**Figure 14:**
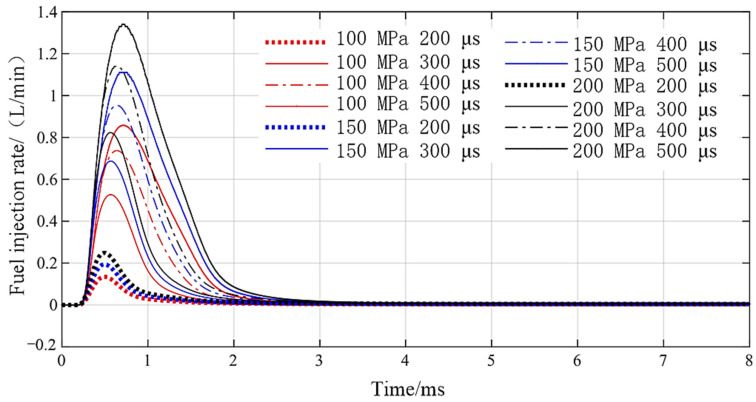
DD-GMI injection rate under different short pulse widths.

Setting the voltage amplitude to 24 V, rail pressure to 100 MPa, 150 MPa, and 200 MPa and pulse width to 200 µs, 300 µs, 400 µs, and 500 µs, the DD-GMI injection rate curve, as shown in Figure 14, is obtained. With the rail pressure increasing from 100 MPa to 200 MPa, the fuel injection rate increases, and the rising speed and falling speed of the fuel injection rate increase, and the time when the fuel injection rate reaches the maximum value is basically the same under different rail pressures. When the pulse width is extended from 200 µs to 500 µs, the fuel injection rate increases, and the rising speed and falling speed of the fuel injection rate also increases. When the pulse width is 200 µs, the maximum injection rate of DD-GMI under different rail pressures is 0.11L/min, 0.18 L/min, and 0.25 L/min, respectively; that is, the shortest injection pulse width of DD-GMI is about 200 s, i.e., DD-GMI has a fast response speed, which makes it possible for the injector to realize multiple injections in the cycle time.

### 4.3. Multiple Injection

**Figure 15:**
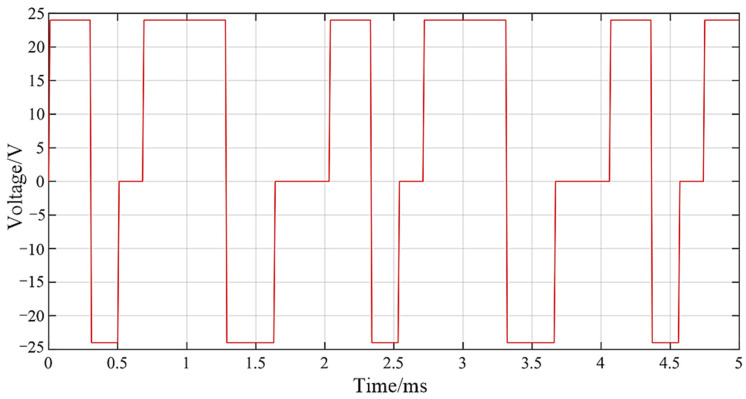
Multiple injection driving waveform.

**Figure 16:**
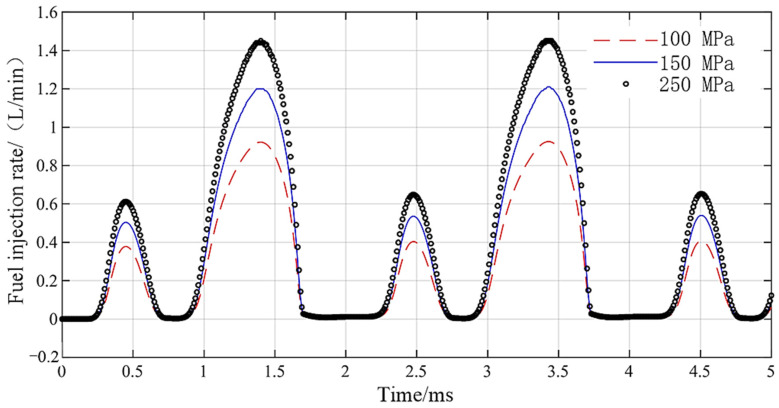
Fuel injection rate of DD-GMI driven by multiple injection driving waveforms.

The driving waveform is shown in Figure 15. The continuous pulse width of forward loading voltage is 300 µs and 600 µs, the pulse width of reverse loading voltage is 180 µs and 300 s, and the amplitude of driving voltage is 24 V. The DD-GMI injection rate curve is shown in Figure 16. The fuel injection rate curve lags behind the voltage driving signal, and the short pulse width and long pulse width of the driving waveform correspond to the small peak and the big peak of the fuel injection rate curve, respectively. With the rail pressure increasing from 100 MPa to 200 MPa, the maximum injection rate of DD-GMI increases from 0.91 L/min to 1.48 L/min. DD-GMI completed five complete injection processes in 5 ms, and the boundary between the two injections was obvious, which indicated that DD-GMI had good multiple injection ability.

## 5. Conclusions

Based on the structure of direct-drive injector and GMM with excellent output performance, the prototype of direct-drive GMI was manufactured, and the AMESim simulation model of GMI was built. The fuel injection performance of GMI was studied and analyzed by means of experiment and simulation.

1. The simulation model of DD-GMI was built. According to the different hydrodynamic characteristics, DD-GMI cavity–pipeline–valve part model was divided into cavity model, pipeline model, valve model, leakage module model, and throttle model for modeling. This part model was combined with the GMA model to complete the integrated modeling of DD-GMI.
2. The experimental verification of DD-GMI integrated model was carried out. When the driving pulse width was larger than 650 µs, the relative error between DD-GMI integrated model and experimental results was less than 5%. By changing the driving voltage, the single injection quantity of DD-GMI under different driving pulse widths (≥650 µs) was tested. The relative error between the model calculation results and the experimental test results was less than 5%, and when the driving pulse width is 2000 µs and the voltage amplitude was 80V, the relative error between them was able to reach 1%. The results show that the model can accurately describe the fuel injection performance of DD-GMI. At the same time, the minimum injection quantity measured by the injector is about 5.9 mm^3^, which shows good injection accuracy.
3. Based on DD-GMI integrated model, fuel injection performance was analyzed. With the increase of driving pulse width, the duration of fuel injection was prolonged, and under the same rail pressure, the rising speed and falling speed of fuel injection rate were basically the same, which indicates that DD-GMI has good working stability. The shortest injection pulse width of DD-GMI was about 200 µs, and its response speed was fast. In 5 ms, DD-GMI completed five complete injection processes by using the waveform drive of short and long square waves and has good multiple injection ability.

## Figures and Tables

**Table 1:** Comparison of material characteristics.

Material	Giant Magnetostrictive Materials	Piezoelectric Ceramic Material	Traditional Magnetostrictive Material
	Terfenol-D	PZT	Ni
Saturated magnetostriction coefficient [*λ*_S_]/(ppm)	1500~2000	100~600	−35~40
Magnetic coupling coefficient [*K_33_*]	0.7~0.72	0.45~0.72	0.3
Energy density [ω]/(kJ·m^−3^)	30~50	0.23~1.0	0.03
Workplace (V/cm) or (kA/m)	~800	124	0.1
Young’s modulus [*E*]/(GPa)	25~35	4.6~6.0	21
Curie point [*T_c_*]/(℃)	380	40~180	376

**Table 2:** Main elements used in the AMESim model.

Simulation Model Icon	Explanation
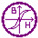 ; 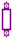 ; 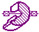 ; 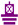 ; 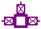 ; 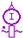 ; 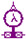 ; 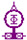	Material defined by parameter settings; basic magnetoresistive unit; radial magnetoresistive unit; zero magnetic potential node; three-terminal magnetic circuit connector; ammeter; magnetic potential sensor; magnetic flux sensor
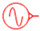 ; 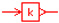 ; 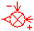 ; 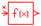 ;	Signal source; gain; three-terminal subtractive connector; convention function
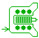 ; 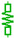 ; 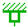 ; 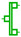 ; 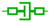 ; 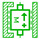	Electromagnetic force function; ideal mechanical spring; zero velocity source; linear translation node that transfers velocity and displacement; damper; mass block with configurable dead center
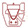 ; 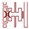 ; 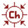 ; 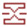	Nozzle; leakage and viscous damping; hydraulic chamber; hydraulic port

**Table 3:** Main parameters of GMA.

	Parameter [Symbol]/Unit	Value		Parameter[Symbol]/Unit	Value
Size parameters	Enameled wire diameter [*φ*]/mm	0.6	Mechanical parameters	Saturation magnetostrictive strain [*λ*_S_]	800
	GMM cylinder length[*l*]/mm	160		GMM bulk density [*ρ*]/(kg/m^3^)	9500
	GMM cylinder cross-sectional area[*A*]/mm^2^	48		GMM damping [*C*_D_]/(N·s/m)	700
	Coil winding inner diameter [*R*_a_]/mm	6		GMM elastic modulus [*E*]/(N/m ^2^)	3 × 10^10^
	Coil winding outer diameter [*R*_b_]/mm	11.5		“T” plunger mass [*m*_2_]/g	23
	Coil winding length [*L*_c_]/mm	160		Spring rate [*k*_2_]/(N/m)	3 × 10^5^
				Equivalent damping [*c*_2_]/(N·s/m)	200
Magnetic parameters			Circuit parameters		
	Pinning loss factor [*k*]/(A/m)	1300			
	Shape parameter [*a*]/(A/m)	9800		Coil turns [*N*]	2000
	Domain Wall Interaction Coefficient [α]	−1.0 × 10^−3^			
	Reversible coefficient [*c*]	0.28		Equivalent inductance [*L*]/mH	8.4
	Saturation magnetization [*M*_S_]/(A/m)	8.0×10^5^		Equivalent resistance [*R*]/mΩ	8.7

## Data Availability

Not applicable.
